# Effectiveness of a Mobile App (PIMPmyHospital) in Reducing Therapeutic Turnaround Times in an Emergency Department: Protocol for a Pre- and Posttest Study

**DOI:** 10.2196/43695

**Published:** 2023-05-03

**Authors:** Frederic Ehrler, Carlotta Tuor, Robin Rey, Rémy Trompier, Antoine Berger, Michael Ramusi, Delphine S Courvoisier, Johan N Siebert

**Affiliations:** 1 Division of Medical Information Sciences Department of Radiology and Medical Informatics Geneva University Hospitals Geneva Switzerland; 2 Faculty of Medicine University of Geneva Geneva Switzerland; 3 Information Technology Department Geneva University Hospitals Geneva Switzerland; 4 Quality of Care Unit Geneva University Hospitals Geneva Switzerland; 5 Geneva Children’s Hospital Department of Pediatric Emergency Medicine Geneva University Hospitals Geneva Switzerland

**Keywords:** clinical laboratory information systems, laboratory results, digital technology, emergency department, emergency service, hospital, length of stay, mobile app, mobile health, mHealth, pediatrics, therapeutic turnaround time

## Abstract

**Background:**

Delays in reviewing issued laboratory results in emergency departments (EDs) can adversely affect efficiency and quality of care. One opportunity to improve therapeutic turnaround time could be to provide real-time access to laboratory results on mobile devices available to every caregiver. We developed a mobile app named “Patients In My Pocket in my Hospital” (PIMPmyHospital) to help ED caregivers automatically obtain and share relevant information about the patients they care for including laboratory results.

**Objective:**

This pre- and posttest study aims to explore whether the implementation of the PIMPmyHospital app impacts the timeliness with which ED physicians and nurses remotely access laboratory results while actively working in their real-world environment, including ED length of stay, technology acceptance and usability among users, and how specific in-app alerts impact on its effectiveness.

**Methods:**

This single-center study of nonequivalent pre- and posttest comparison group design will be conducted before and after the implementation of the app in a tertiary pediatric ED in Switzerland. The retrospective period will cover the previous 12 months, and the prospective period will cover the following 6 months. Participants will be postgraduate residents pursuing a ≤6-year residency in pediatrics, pediatric emergency medicine fellows, and registered nurses from the pediatric ED. The primary outcome will be the mean elapsed time in minutes from delivery of laboratory results to caregivers’ consideration by accessing them either through the hospital’s electronic medical records or through the app before and after the implementation of the app, respectively. As secondary outcomes, participants will be queried about the acceptance and usability of the app using the Unified Theory of Acceptance and Use of Technology model and the System Usability Scale. ED length of stay will be compared before and after the implementation of the app for patients with laboratory results. The impact of specific alerts on the app, such as a flashing icon or sound for reported pathological values, will be reported.

**Results:**

Retrospective data collection gathered from the institutional data set will span a 12-month period from October 2021 to October 2022, while the 6-month prospective collection will begin with the implementation of the app in November 2022 and is expected to cease at the end of April 2023. We expect the results of the study to be published in a peer-reviewed journal in late 2023.

**Conclusions:**

This study will show the potential reach, effectiveness, acceptance, and use of the PIMPmyHospital app among ED caregivers. The findings of this study will serve as the basis for future research on the app and any further development to improve its effectiveness. Trial Registration: ClinicalTrials.gov NCT05557331; https://clinicaltrials.gov/ct2/show/NCT05557331

**Trial Registration:**

ClinicalTrials.gov NCT05557331; https://clinicaltrials.gov/ct2/show/NCT05557331

**International Registered Report Identifier (IRRID):**

PRR1-10.2196/43695

## Introduction

### Background

Emergency department (ED) overcrowding is a global health problem [1], leading to prolonged ED length of stay (ED-LOS). ED-LOS is the time interval between the patient’s arrival in the ED for registration or triage to disposition from the ED, either to be admitted to a hospital, discharged home, or transferred to another care setting. ED-LOS is considered as a key indicator of the performance, quality, and operational efficiency of an ED and, more broadly, the entire hospital to which the ED belongs [2]. It also serves as a benchmark metric between institutions. Prolonged ED-LOS, that is, stays exceeding 6 hours (with cutoffs ranging from 4 to 48 hours [3]), can adversely affect efficiency and quality of care, patient outcomes, and patient satisfaction [4].

According to the conceptual input-throughput-output model proposed by Asplin et al [5], ED congestion is partitioned into 3 interdependent components in terms of patient flows into, through, and out of the ED, respectively. The entire laboratory testing process, also known as therapeutic turnaround time (TAT) and part of the throughput component, is a major contributor to prolonging ED-LOS [6,7]. Laboratory tests are ordered in nearly 48% of ED visits [8], and 70% of patient-management decisions are based on these tests [9]. Numerous mitigation strategies to minimize the intralaboratory analytic time, such as laboratory automation and digitalization, have been implemented [10,11]. Yet, it is estimated that up to 96% of the delays that contribute to the therapeutic TAT occur in the pre- and postanalytical phases outside of the central laboratory [12,13]. In the latter case, caregivers’ delayed awareness and timely review of available laboratory results have been described as the largest component of perceived therapeutic TAT [11], strongly contributing to prolonged hospital LOS [14]. This delay may be partly related to the fact that busy, multitasking ED physicians and nurses are cognitively distracted from reviewing laboratory results in a timely manner in the absence of individualized, automated real-time prompts. Prompt awareness of results’ availability in real time requires health care personnel to regularly access electronic medical records (EMR), leading to incessant and time-consuming trips to computer workstations, thus distracting them from patient care in an extremely challenging and changing environment.

### Previous Work

Targeted interventions to reduce the postanalytical TAT phase may contribute to shortening ED-LOS, but mobile digital health solutions to achieve this goal are scarce. In a previous study [15], we described the user-centered development and early technology acceptance evaluation of a mobile app called “Patients In My Pocket in my Hospital” (*PIMPmyHospital*). The app was designed to help ED caregivers obtain relevant information in real time for the patients they care for, including laboratory and imaging results. The app also offers an end-to-end encrypted chat to provide a remote communication channel between caregivers caring for the same patients. In a randomized controlled pilot trial [16], the time required for ED physicians and nurses to review laboratory results after their release was significantly reduced by 92% using the app compared to the usual procedure in a semisimulated model. However, the potential effectiveness of this app in mitigating ED-LOS by taking cognizance of laboratory results in a shorter time frame than usual remains to be evaluated in daily clinical ED practice.

### Objectives

In this study, we present the protocol for a pre- and posttest study. Its objective is to evaluate whether the use of the *PIMPmyHospital* app alters ED physicians’ and nurses’ temporal efficiency to access laboratory results more quickly while actively working in an ED. Temporal efficiency will be assessed by measuring the time between the release of the laboratory results in the clinical information system and their acknowledgment by these clinicians. To our knowledge, no app that can access laboratory results in real time in the ED has been described to date [17]. The study will be structured according to the “Population,” “Intervention,” “Comparison,” and “Outcome” framework as follows: (1) participants: medical and nursing staff working in the pediatric ED; (2) intervention: implementation of the mobile app in the pediatric ED; (3) comparison: remote access to laboratory results in the pediatric ED through the app versus standard access to laboratory results prior to its implementation; (4) outcomes: times to access laboratory results, ED-LOS, technology acceptance, and app’s usability among users, and how specific in-app alerts impact on its effectiveness.

## Methods

### Study Design

We will conduct an 18-month, single-center, nonequivalent comparison group, pre-post study. We will compare the control and experimental groups on outcome measures before (12-month period) and after (6-month period) the implementation and use of the app. This study protocol adheres to the Standard Protocol Items: Recommendations for Interventional Trials 2013 Checklist [18]. All data will be deidentified. The study will be conducted according to the principles of the Declaration of Helsinki [19] and Good Clinical Practice guidelines [20]. It is our intention to present these at scientific congresses and to publish the results in an international peer-reviewed journal, irrespective of the magnitude or direction of effect. Only deidentified data will be available to researchers for analysis. The study is registered at ClinicalTrials.gov on September 28, 2022 (NCT05557331).

### Participants

Eligible participants will be postgraduate residents pursuing a less than 6-year residency in pediatrics, pediatric emergency medicine fellows, and registered nurses from the pediatric ED (aged >18 years). Written informed consent will be obtained from each participant in the experimental group after full information disclosure prior to study participation. On the first day, after completing the informed consent form, participants will be asked to download the app to their personal smartphone through the secure institutional Microsoft Intune portal (Microsoft Corporation). They will then be required to attend a unique standardized 5-minute introduction course to the app and its functionality by the study investigators, but they will not receive specific instructions on how often to use the app. The participants will remain the same throughout the intervention period. No one will be asked to advise on the interpretation of the results.

### Setting

The study will take place in the pediatric ED of a tertiary referral and major trauma hospital in Switzerland, with an annual volume of 35,000 visits. This should represent approximately 16,600 visits over the 6-month prospective intervention period, of which approximately one-half could potentially benefit from laboratory tests. The ED has 14 centrally monitored treatment rooms, a resuscitation room, a 5-bed short-term observation room (ie, <24 hours), a plaster room, a hypostimulation room, and a dedicated interview room. The patient population consists of local children from birth to 16 years of age with medical, surgical, and traumatology emergencies, as well as transfer cases from other hospitals in Switzerland or children located in neighboring France.

The usual number of residents is 10 on a monthly basis during a 4-month period. They provide coverage for 6-7 overlapping shifts, which begin at different times, with each shift ranging from 10 hours (9 hours of patient care, 1 hour of recovery) to 12 hours (12 hours of patient care, no recovery time at night) on weekdays and 6 shifts on weekends to cover a 24-hour day (Table 1). During the intervention schedule, residents typically work 3-4 night shifts followed by 72 hours off, then 3-5 consecutive day shifts before 2 days off, followed by 2-day shifts. This pattern repeats throughout the month, with occasional additional days off or vacations. The 14 fellows (some of whom are part-time) work in an irregular combination of 6 overlapping 8-12 hour shifts on weekdays and 3 shifts on weekends (Table 1). Finally, 17 nurses out of a total of 43 cover a 24-hour day. This represents a total coverage of 70-78 hours, 30-48 hours, and 102 hours per day for residents, fellows, and nurses, respectively. The ED uses the Canadian Triage Acuity Scale [21] to guide patient treatment based on medical-surgical urgency. On average, each resident sees about 300 patients per month and fellows 50-70, with a ratio of medicine: surgery-trauma cases of 2/3:1/3. The standard physician-to-patient ratio ranges from 1:5 to 1:7 and varies according to disease acuity and time of the visit. The nurse-to-patient ratio ranges from 1:3 to 1:5. Caregivers see patients on a first-view [22], on-site, 24/7 basis. Whether they work 8-, 10-, or 12-hour shifts, they all work in the same area, have access to the same patient volume, and see the same patient complaints. They have full, secure 24/7 electronic access to laboratory results.

**Table 1:** Work shifts by time of day and occupation.

Caregiver and work shift		Count, n	
		Weekdays	Weekend
**Residents**			
	7:45-17:45	1	0
	8:00-20:00	1	2
	10:00-22:00	1	1
	14:00-24:00	1	0
	16:00-2:00	1	1
	20:00-8:00	1	1
	21:00-9:00	1	1
**Fellows**			
	7:30-16:30	1	0
	8:00-18:00	1	0
	8:00-20:00	0	1
	14:00-20:00	1	0
	14:30-23:00	1	0
	15:30-24:00	1	0
	20:00-8:00	1	1
**Nurses**			
	7:00-15:30	5	5
	11:30-20:00	1	1
	15:00-23:30	6	6
	23:00-7:15	5	5

### The PIMPmyHospital Mobile App

Information regarding the app has been published previously [16]. In brief, the app was developed iteratively following a user-centered approach starting with the identification of the main functionalities that meet end-user needs [15]. The system operates on a client-server architecture. Four microservices developed on the Java Spring Boot framework ensure (1) transmission of patient information, (2) access to real-time laboratory results, (3) the trigger of push notifications, and (4) exchange of information between caregivers through an instant messaging system. These microservices exchange information with the front end developed in the Angular and Ionic frameworks through a restful app programming interface. Patient medical data are not stored on the app itself, but in the institution’s EMR on secure servers to which the app connects. The exchange of data between the app and these servers is secured by the use of JSON web tokens obtained during the authentication of caregivers through a Keycloak identity and access management solution. User authentication on the device relies both on a login password and a specific certificate installed on the user’s device. The app runs on Google’s Android and Apple’s iOS operating systems.

The app’s interface features a home page with a personalized view of the patients that each caregiver is taking care of, together with associated information (eg, patient identity, time since admission, and patient’s geospatial location within the ED) and clickable actions next to them [16]. In particular, dropdown contextual tabs provide real-time access to laboratory results retrieved from the hospital patient’s EMR. Visual push notifications occur as soon as new results are made available. The entire patient management process is available to the caregiver in the palm of their hand and can be accessed at any time while on the go, hence the app’s name “Patients in my Pocket.”

### Procedure and Intervention

**Figure 1:**
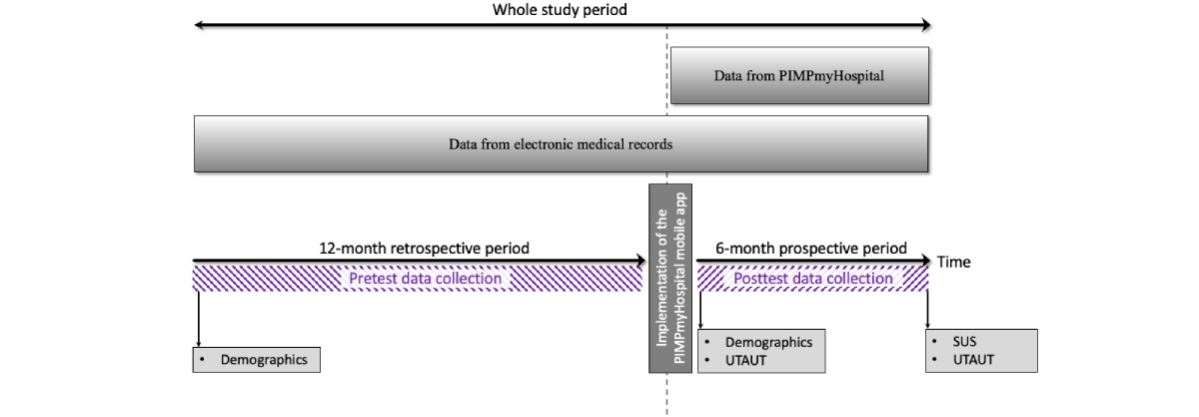
Study procedure. PIMPmyHospital: Patients In My Pocket in my Hospital; SUS: System Usability Scale; UTAUT: Unified Theory of Acceptance and Use of Technology.

This is a pre-post study. Before the implementation of the app, EMR-based data from the past 12 months will be collected retrospectively. These data will include the times (ie, HH:MM:SS) that results issued by the central laboratory were available on the institutional EMR as well as the times at which these results were accessed by caregivers through conventional computerized workstations. After the implementation of the app, data will be collected prospectively over a 6-month period of use. This will include the times (HH:MM:SS) when the results issued by the central laboratory are made available through the app on caregivers’ smartphones and access times as well as concurrent or preferred access times on the conventional computerized workstations should this occur. Individual acceptance of the app on the first day (a priori) and then on the last day of the intervention will be evaluated through the Unified Theory of Acceptance and Use of Technology (UTAUT) [23]. Usability of the app will be measured by the System Usability Scale (SUS) [24]. Figure 1 shows the study procedure, and Table 2 presents the study schedule. Use of the app will be on a 24-hour basis and at the discretion of participants.

**Table 2:** Study schedule.

		Study period and time point			
		Pretest (*–t*_12__)_	Enrolment/app implementation (*t*_0_)	Posttest (*+t*_6_)	Closeout
**Study procedures**					
	Recruitment		✓^a^		
	Eligibility screening		✓		
	Informed consent form		✓		
**Assessments**					
	Case report form (demographics)		✓		
	EMR^b^-based data	✓	✓	✓	
	App-based data		✓	✓	
	UTAUT^c^		✓		✓
	SUS^d^				✓

^a^✓: Performed.

^b^EMR: electronic medical record.

^c^UTAUT: Unified Theory of Acceptance and Use of Technology.

^d^SUS: System Usability Scale.

### Technology Acceptance Terminology and Definition

We use technology acceptance to refer to an initial acceptance of use, as recently defined by Nadal et al [25], to explicitly distinguish between the different temporal stages of technological acceptance. It refers to users’ first interactions with the app at the preadoption stage before sustained acceptance of its use in the postadoption stage.

### Outcomes and Measures

#### Primary Outcome: Time to Laboratory Access

The primary outcome will be the mean elapsed time in minutes between the delivery of the laboratory results and the caregiver accessing them, both before and after the implementation of the app.

#### Secondary Outcome: Technology Acceptance and Usability

The secondary outcomes will be the technology acceptance and usability of the app. The 8-construct, 31-item UTAUT [23] will be used to measure app acceptance and intention to use. This is a 5-point Likert-type scale (1: strongly disagree, 2: moderately disagree, 3: neutral [neither disagree nor agree], 4: moderately agree, and 5: strongly agree). Usability of the app will be measured using the SUS designed by Jordan [24]. According to the International Organization for Standardization, the SUS assesses effectiveness (ie, the ability of users to use the product), efficiency (ie, the effort to use the product), and satisfaction (ie, how the users felt when using the product). It comprises a 10-item questionnaire with 5 response options for each item based on their level of agreement, ranging from 1 (strongly disagree) to 5 (strongly agree). According to the scoring system by Jordan [24], for odd-numbered (1, 3, 5, 7, and 9) statements (the positively-worded items), the score contribution is equal to the scale position minus 1 (eg, strongly agree: 5–1=4). For even-numbered (2, 4, 6, 8, and 10) statements (the negatively worded items), the score contribution is equal to 5 minus the scale position (eg, strongly agree: 5–5=0). Each score contribution falls within the range of 0 to 4. Participants’ scores for each item are then added up together and multiplied by 2.5 to convert the original scores of 0-40 to 0-100. Although the scores range from 0 to 100, these are not percentages of usability. The higher the score, the better the usability. To obtain a SUS score of 100, the respondent must answer 5 to all odd questions and 0 to all even questions. The original SUS items are presented in Multimedia Appendix 1. When translating the SUS questionnaire from its original version to French [26], we replaced the general term “system” with the specific term *PIMPmyHospital*. Another secondary outcome will be the mean ED-LOS per patient measured from the point when patients are triaged in the ED to discharge from the ED, defined as patients leaving the ED, whether admitted to the intensive care unit or ward, transferred to another hospital for admission, discharged home, or those who left without being seen. These data will be extracted from the hospital administrative electronic database. Finally, we will determine whether specific alerts, such as a flashing icon or sound for reported pathological values of laboratory results, have an impact on the results.

### Data Transfer, Safety, and Privacy

Access to the app is controlled through a 2-factor authentication process. An enterprise environment is created on the health care smartphone using the Microsoft Intune software (Microsoft Corp.), for both Android and iOS operating systems. This environment ensures that the app is run in a secure context where only an authorized app can be installed. This environment also enables the installation of a certificate that serves as a second authentication factor, while the first factor is the user’s login and password. Access rights are managed by the Active Directory group, which enables access to ED caregivers only. All data collected by the app are transmitted securely through a hypertext secure transfer protocol and stored exclusively on the hospital’s secured internal server.

### Data Collection

Electronic data will be extracted from the institutional data lake. The data lake stores all data and events from the EMR information system in a database and can be retrieved using Simple Protocol and RDF Query Language (SPARQL) queries. In the preintervention period, the time stamp of the laboratory results on the EMR will be identified as the time of issuance of the said results. Access to these results by the physicians and nurses will be defined by the time stamp of the first access to the laboratory results’ viewer on the EMR. Access to results on the app will be computed either by the time stamp of the push notification access or to the dedicated page on the app. No other patient-level data such as identifiers or clinical characteristics will be collected. Collected data will be transmitted to the REDCap database web app (REDCap, Vanderbilt University) hosted by the hospital for analysis.

### Blinding

Although neither the participants nor the study investigators can be blinded to the intervention, both will be blinded to the results of the study until their final analysis by the statistician. Participants will be informed only of the overall purpose of the study, which is to evaluate the effects of the app on quality of care, but not about the aim of reducing the time to access laboratory results in order to minimize bias. The statistician will remain blinded to allocation until the final analysis.

### Sample Size

Physicians in the pediatric ED see on average 900 patients over 3 months. Assuming an intraclass correlation of 0.1 in time between delivery of the laboratory results and the caregiver accessing them, 5253 individual patients are required to have a 90% chance of detecting, as significant at the 5% level, a decrease in a time difference of 0.5 SD. This represents approximately 18 physicians including 100 patients per month over 3 months. In order to obtain 18 physicians, we will recruit physicians over 2 consecutive rotation periods.

### Statistical Analysis

The association of app use with the time between delivery of the laboratory results and their access by caregivers will be assessed using generalized estimating equations with an exchangeable correlation structure to account for the nested structure of the outcome (ie, time) within the physicians. To account for potential differences in case-mix and physician characteristics, we will use multivariable generalized estimating equations. The covariates will be as follows: (1) the triage acuity scale, (2) type of pathology (medical, surgical-traumatological, psychiatric), (3) experience of the caregiver expressed in years since certification, (4) patient age, (5) patient sex, (6) workload represented by the number of patients present in the emergency room, (7) time of day or night, (8) day of the week, and (9) month of the year (seasonal variability). To account for the possible effect of exogenous factors that would impose an asymmetry in the data (skewness on the right) by extending the awareness of laboratory results, we will identify these factors as the study progresses prospectively and take them into account in the final analysis. The same analyses will be performed for the nurses. We will carefully monitor and evaluate through descriptive analyses any differences between nurses’ and physicians’ frequency access to laboratory results. If the target sample size cannot be reached, we will first extend the prospective data collection period by 3 months (deemed ethically acceptable as not impacting patients) or present only the results of qualitative user experience interviews.

For the technology acceptance self-administered survey questionnaire, scores on the 8 technology acceptance constructs will be described using descriptive statistics. Items on the SUS questionnaire will be described using frequencies. Analyses will be carried out using R software (R Core Team) [27]. All statistical tests will be 2-sided, with an α risk of .05.

### Ethics Approval

The study protocol received a declaration of no objection by SwissEthics on October 18, 2022 (Req-2022-01156), which stated that the need for approval was waived as its purpose was to examine the effect of the intervention on health care providers and thus it does not fall within the scope of the Swiss Federal Law on Human Subject Research, with a waiver of written informed consent.

Data available to research team members will only contain deidentified, coded information. All records that contain personal identifiers, such as consent forms and UTAUT and SUS questionnaires, will be stored separately from study records. Each participant file will be identified by a code number. Study data will not be released to any other individual outside the research team. Only deidentified data will be published and available to researchers for analysis. There will be no financial compensation or incentive for participants.

### Dissemination Policy

Only study investigators will have the right to access and analyze the data for dissemination purposes. The principal investigator will oversee dissemination activities to ensure that team members with the necessary subject matter expertise are involved in every aspect of dissemination. We plan to disseminate the results of this study through peer-reviewed publications, conferences, as well as other avenues as appropriate, regardless of the findings.

## Results

Retrospective laboratory data will be arbitrarily collected from October 31, 2021, to October 31, 2022. Prospective data will be collected from the start of enrollment on November 1, 2022, through April 30, 2023. We anticipate an analysis of the results by mid-2023 and expect the results to be published in late 2023.

## Discussion

### Strengths and Perspectives With the Existing Literature

To the best of our knowledge, there are no previous studies describing the effectiveness of a mobile app to provide real-time mobile access to laboratory results in EDs [17]. This study protocol is the first to propose the exploration of the potentially beneficial effect of such an app on the reduction of therapeutic TAT and its acceptance and usability by end users. The study investigators anticipate that implementation of the app will shorten times to consideration of laboratory results among participants who use it compared to those who have not used it previously. We plan to generate a stronger evidence base for a broader implementation of dedicated mobile apps for remote, real-time access to laboratory results in EDs. We also expect good acceptance of the app as well as good usability. One of the strengths of this study is the use of an app whose iterative design and development involved a multidisciplinary end user–centered approach [15]. In addition, the app’s ability to reduce the time taken by pediatric ED caregivers to consider laboratory results has already been the subject of a randomized controlled pilot trial in a semisimulated ED environment [16]. This study will attempt to verify its encouraging results in a real environment with its spatial and temporal constraints and inherent human factors.

In terms of implications, the use of dedicated mobile apps could represent a paradigm shift where information would come directly and synchronously to caregivers rather than having to travel to retrieve it. A shorter TAT for the postanalytical phases could be associated with a shorter ED-LOS and thus improve the quality of care and patient satisfaction. Moreover, such a solution could be extended to other care units where the use of mobile apps would make it possible to get rid of the distances and delays to access laboratory results.

### Limitations

This study has some limitations. First, participants who agree to be prospectively enrolled may be interested or engaged in digital health promotion in general and mobile technologies in particular, influencing their a priori motivation to use the app. If so, this should be reflected in the UTAUT questionnaire, which is an outcome of interest. Second, the study will be conducted in a pediatric ED, which may limit the generalizability of the findings to adult EDs. For example, the acuity of age-specific diseases may imply a different awareness in the consideration of laboratory results. Other limitations may be identified during the study itself and will be noted after their identification.

### Conclusions

Our intervention can serve as a foundation for a broader implementation of supportive mobile apps for in-hospital caregivers to improve the timely availability of EMR-derived results and the efficiency of care by reducing therapeutic TAT and ED-LOS.

## Appendix

Multimedia Appendix 1 The System Usability Scale. Adapted from Jordan [24].

## Abbreviations

ED: emergency department
ED-LOS: emergency department length of stay
EMR: electronic medical record
PIMPmyHospital: Patients In My Pocket in my Hospital
SUS: System Usability Scale
TAT: therapeutic turnaround time
UTAUT: Unified Theory of Acceptance and Use of tTechnology

## Data Availability

Data sharing is not applicable to this paper as no data were generated or analyzed during the preparation of this protocol. The final data sets established and analyzed during the study will not be publicly available. An anonymous copy of the final data sets underlying publications resulting from this study will be available from the corresponding author upon reasonable and approved request.
