# Supplementary material for: Effectiveness of a Mobile App (PIMPmyHospital) in Reducing Therapeutic Turnaround Times in an Emergency Department: Protocol for a Pre- and Posttest Study
Source: JMIR Res Protoc. 2023 May 3;12:e43695. doi: 10.2196/43695 (PMC10193207; doi:10.2196/43695)
Supplement: Multimedia Appendix 1 [file resprot_v12i1e43695_app1.docx]

Q1: I think that I would like to use *PIMPmyHospital* frequently

Q2: I found *PIMPmyHospital* unnecessarily complex

Q3: I thought *PIMPmyHospital* was easy to use

Q4: I think that I would need the support of a technical person to be able to use *PIMPmyHospital*

Q5: I found that the various functions in *PIMPmyHospital* were well integrated

Q6: I thought that there was too much inconsistency in *PIMPmyHospital*

Q7: I would imagine that most people would learn to use *PIMPmyHospital* very quickly

Q8: I found *PIMPmyHospital* very cumbersome to use

Q9: I felt very confident using *PIMPmyHospital*

Q10: I needed to learn a lot of things before I could get going with *PIMPmyHospital*
